# The Effects of Daridorexant on Patients With Comorbid Insomnia Disorder and Untreated Mild Obstructive Sleep Apnoea: A Post Hoc Subgroup Analysis of a Phase 3, Randomised Clinical Trial

**DOI:** 10.1111/jsr.70135

**Published:** 2025-07-14

**Authors:** Christopher J. Lettieri, Orestis Briasoulis, Damien Léger, Pierre-Philippe Luyet, Jean-Louis Pépin, Stuart F. Quan, Janna Raphelson, Paul Saskin, Atul Malhotra

**Affiliations:** 1 Idorsia Pharmaceuticals US Ltd, Radnor, Pennsylvania, USA; 2 Pulmonary, Critical Care and Sleep Medicine, Department of Medicine, Uniformed Services University, Bethesda, Maryland, USA; 3 Idorsia Pharmaceuticals Ltd, Allschwil, Switzerland; 4 VIFASOM (Vigilance Fatigue et Santé Publique), Université Paris Cité, Paris, France; 5 APHP, Hôtel Dieu de Paris, Centre du Sommeil et de la Vigilance, Paris, France; 6 Grenoble Alpes University, Grenoble, France; 7 Division of Sleep and Circadian Disorders, Department of Medicine, Brigham and Women’s Hospital and Division of Sleep Medicine, Harvard Medical School, Boston, Massachusetts, USA; 8 Division of Pulmonary and Critical Care Medicine, Department of Medicine, University of California, San Diego, San Diego, California, USA

**Keywords:** Daridorexant, dual orexin receptor antagonist, insomnia disorder, obstructive sleep apnoea

## Abstract

Daridorexant, a dual orexin receptor antagonist, is approved for the treatment of insomnia disorder in adults. Approximately 30%–35% of patients with insomnia disorder also have obstructive sleep apnoea (OSA) of any severity. It is unclear whether sleep medications provide safe and effective treatment for insomnia in these patients. This post hoc analysis evaluated the efficacy and safety of daridorexant 25 and 50 mg on objective and self-reported insomnia variables and self-reported daytime functioning in patients with untreated mild OSA and comorbid insomnia disorder (COMISA). This analysis included participants with insomnia disorder enrolled in the Phase 3 study assessing either daridorexant 25 or 50 mg with an apnoea/hypopnoea index 5–< 15 events/h (‘mild OSA’). Wake after sleep onset (WASO), latency to persistent sleep (LPS), self-reported total sleep time (sTST) and the Insomnia Daytime Symptoms and Impacts Questionnaire (IDSIQ) were assessed at Months 1 and 3. Safety endpoints were treatment-emergent adverse events, daytime somnolence and next-morning residual effects. In participants with mild OSA, daridorexant improved WASO, LPS, sTST and IDSIQ total score over time. The average treatment effect size for all efficacy parameters was numerically greater with daridorexant 50 mg than with daridorexant 25 mg; daridorexant 25 mg was not always greater than placebo. No safety concerns were reported for daridorexant 50 or 25 mg. In participants with comorbid insomnia and untreated mild OSA, daridorexant 50 mg versus placebo improved all sleep parameters over time and was well tolerated. Daridorexant warrants further investigation in COMISA.

**Trial Registration:**

ClinicalTrials.gov identifier: NCT03545191

## 1 | Introduction

Insomnia disorder and obstructive sleep apnoea (OSA) are two of the most prevalent sleep disorders and they often coexist (Cho et al. 2018; Sweetman et al. 2019, 2023). Recent epidemiology studies have estimated that ~10% of the adult population is affected by insomnia disorder (Morin and Jarrin 2022) and that ~30%–35% of these also have OSA (Zhang et al. 2019; Sweetman et al. 2021; Riemann et al. 2023; Guilleminault et al. 1973). Comorbid insomnia and sleep apnoea (COMISA) pose a major public health problem worldwide (Morin and Jarrin 2022; Riemann et al. 2023). Patients with COMISA may have greater daytime impairments, reduced quality of life and a higher risk of cardiovascular disease than those with either disorder alone (Cho et al. 2018; Sweetman et al. 2023; Zhang et al. 2019; Lechat et al. 2022; Peppard et al. 2000; McNicholas et al. 2007).

It is unclear whether treatments for insomnia disorder are safe and effective in patients with COMISA (Zhang et al. 2019). Many sleep medications require cautionary use in patients with OSA as they can impair nighttime respiration or exacerbate common symptoms of insomnia and OSA such as daytime somnolence (Mason et al. 2015; AMBIEN (zolpidem tartrate) 2022; ATIVAN (lorazepam) 2023). Some patients with OSA, especially those with COMISA, have a low arousal threshold (propensity to wake up) and may be particularly challenging to treat as they can develop insomnia with the application of positive airway pressure (PAP) therapy (Edwards et al. 2014; Zinchuk et al. 2018). Hence, the desired treatment effects of sleep medications for patients with COMISA include improved sleep onset and maintenance without worsening daytime somnolence and fatigue.

Dual orexin receptor antagonists have emerged as a new treatment for insomnia disorder. They block the binding of orexin A and orexin B to the two orexin receptors and thereby inhibit the pathways responsible for overactive wake signalling in insomnia disorder (Ziemichod et al. 2022). Daridorexant, a dual orexin receptor antagonist, is approved in the United States, EU, United Kingdom, Switzerland, Canada, Japan and Hong Kong for the treatment of insomnia disorder in adults (at doses of 25 and 50 mg) (Mignot et al. 2022; Chakraborty et al. 2023; QUVIVIQ (Daridorexant) 2022a, 2022b, 2022c, 2023, 2024a, 2024b). The latest EU guidelines for insomnia disorder recommend daridorexant as the only drug for both the short- and long-term treatment of individuals affected by chronic insomnia with grade A evidence when cognitive-behavioural therapy (CBT) is not sufficiently effective or available (Riemann et al. 2023).

Daridorexant has a beneficial pharmacokinetic/pharmacodynamic profile promoting rapid absorption to ensure sleep onset and a distribution and elimination which allows maintaining sleep over the entire night while minimising next morning residual effects (Ziemichod et al. 2022; Muehlan et al. 2020). A terminal half-life of 8 h prevents drug accumulation (Ziemichod et al. 2022; Muehlan et al. 2020). In Phase 3 studies performed in participants with insomnia disorder, daridorexant (50 and 25 mg) improved objective sleep onset and sleep maintenance and the participants’ self-reported assessment of sleep quantity (Mignot et al. 2022). Daridorexant 50 mg also improved daytime functioning assessed by the Insomnia Daytime Symptoms and Impacts Questionnaire (IDSIQ) total and domain scores, a validated patient-reported outcome instrument (Mignot et al. 2022; Hudgens et al. 2021). The safety profiles of daridorexant 50 and 25 mg were comparable and showed no physical dependence and no next-morning residual sleepiness, with rates of somnolence and falls comparable to placebo at the highest dose of 50 mg (Mignot et al. 2022). Furthermore, in Phase 1 studies in patients with mild and moderate OSA (*n* = 28) and in patients with severe OSA (*n* = 16) daridorexant 50 mg (for five nights) was not associated with impairment of night-time respiratory function as assessed by the apnoea-hypopnoea index (AHI) or oxygen saturation (Boof et al. 2021, 2023).

The efficacy and safety of daridorexant has not yet been assessed in patients with comorbid insomnia disorder and OSA. The objective of this post hoc analysis of the Phase 3 study that assessed the two approved doses of daridorexant (25 and 50 mg) was to investigate the efficacy of daridorexant on sleep parameters and its safety in the subgroup of participants with comorbid insomnia disorder and untreated mild OSA, who had been undiagnosed before screening.

## 2 | Methods

### 2.1 | Study Design

We conducted a post hoc subgroup analysis of a previously published Phase 3 clinical trial in adult participants with insomnia disorder, which assessed daridorexant at two approved dosage levels (ClinicalTrials.gov identifier NCT03545191) (Mignot et al. 2022).

The study design of the Phase 3 study has been previously described in detail (Mignot et al. 2022). In short, the randomised, double-blind, placebo-controlled, parallel-group trial investigated the efficacy and safety of daridorexant 25 and 50 mg versus placebo in adults with insomnia disorder. The study consisted of a screening period (7–18 days), a single-blind placebo run-in period (13–24 days), followed by a double-blind treatment period (3 months), a single-blind placebo run-out period (7 days) and a 23-day safety follow-up period. Baseline values for sleep parameters and daytime functioning were determined during the placebo run-in period, and endpoints were assessed at Months 1 and 3 of the double-blind treatment period. No major changes were performed to the methods in the original protocol, including the data collection and trial outcomes and there was no planned interim analysis. The study was successfully completed after the required number of participants was recruited and all participants reached the end of study visit.

For the double-blind treatment period, randomisation was stratified by age group (< 65 and ≥ 65 years) with participants receiving daridorexant 25 mg, daridorexant 50 mg or placebo every evening (1:1:1). Participants were assessed by polysomnography (PSG) for two consecutive nights at baseline, Months 1 and 3. The technical requirements for the montage and recording of the PSG followed the recommendations of the American Academy of Sleep Medicine (AASM) guidelines (Berry et al. 2017, 4112). On both nights, the standard PSG montage included six electroencephalogram channels (EEG: F3, F4, C3, C4, O1, O2) referenced to contralateral mastoids (M1, M2), two submental electromyogram (EMG) channels, two electrooculogram (EOG) channels and an electrocardiogram channel (positioned below the R-L clavicle). At screening, to check eligibility, a single PSG night was performed and the montage included the 11 aforementioned channels plus seven additional channels for respiration transducer belts (chest and abdomen), oximeter, thermistor, nasal pressure transducer and tibialis movement sensors (left and right limb). PSG data were evaluated and scored centrally by an independent expert sleep technologist following the AASM guidelines (Berry et al. 2017, 4112).

Throughout the trial, participants completed daily questionnaires to determine scores for the insomnia severity index (ISI), the visual analogue scale (VAS) and the IDSIQ scores.

Of note, AHI data and oxygen saturation assessing OSA severity were collected at screening as eligibility criteria, but not over time.

The Phase 3 study adhered to the Declaration of Helsinki, Good Clinical Practice guidelines, International Council for Harmonisation guidelines and local regulations. The study protocol was approved by the appropriate institutional review boards or independent ethics committees. The full study protocol can be obtained in the supplement of the original publication. All participants provided written informed consent before participation in the study.

### 2.2 | Study Participants

Eligibility criteria for the Phase 3 study have been previously described (Mignot et al. 2022). In short, participants had to be aged 18 years or older with a diagnosis of chronic insomnia disorder according to the Diagnostic and Statistical Manual of Mental Disorders, Fifth Edition (American Psychiatric Association 2013), have an ISI score ≥ 15 (ISI scores range from 0 [no insomnia] to 28 [severe insomnia]), and a self-reported history of disturbed sleep (≥ 30 min to fall asleep, ≥ 30 min awake during sleep time, self-reported total sleep time [sTST] of ≤ 6.5 h) for > 3 nights per week for ≥ 3 months before screening. During the placebo run-in period, these self-reported sleep parameters had to be met on at least three of seven nights. In addition, PSG criteria (latency to persistent sleep [LPS] ≥ 20 min, wake after sleep onset [WASO] ≥ 30 min and total sleep time of < 7 h) had to be met.

Participants with moderate or severe OSA (AHI ≥ 15 events/h) or events associated with oxygen saturation (pulse oximetry) of < 80% at screening PSG were excluded from the study. Patients with a BMI < 18.5 or > 40.0 kg/m2, a history of sleep-related breathing disorders, any sleep disorder other than insomnia or suicide ideation/attempt, self-reported daytime napping (≥ 1 h/day ≥ 3 days/week), acute/unstable psychiatric conditions, alcohol/drug abuse, a periodic limb movement arousal index ≥ 15/h and restless legs syndrome were also excluded. The use of any central nervous system-acting medications (including centrally acting anticholinergics, sedating antihistamines, psychotropics, herbal preparations with possible psychotropic effects, anti-convulsants), within at least 2 weeks prior to screening or five half-lives of the drug (whichever was longer) or moderate/strong CYP3A4 inhibitors or inducers was forbidden until 24 h after end of treatment.

Participants were excluded from the Phase 3 study based on the study protocol’s eligibility criteria if they had moderate or severe OSA (AHI ≥ 15 events/h), events associated with oxygen saturation (pulse oximetry) of < 80% at screening PSG, or any lifetime history of a sleep-related breathing disorder, including chronic obstructive pulmonary disease and sleep apnoea.

Participants with untreated mild OSA (AHI 5–< 15 events/h) were enrolled in line with the study protocol and are the key focus of this post hoc subgroup analysis. This subgroup was not predefined in the study protocol, and randomisation was not stratified based on AHI at screening. Participants with AHI < 5 events/h were categorised as having no OSA (‘without OSA’). Results for the subgroup without OSA are included in the Supporting Information as reference only.

### 2.3 | Efficacy Endpoints

Efficacy measures are reported at baseline, Months 1 and 3, and as change from baseline at Months 1 and 3.

Night-time efficacy endpoints included WASO, LPS and sTST. WASO and LPS values are objective measures and were reported as the mean of PSG recordings obtained over two consecutive nights at baseline, Months 1 and 3. sTST data were obtained from the mean of daily entries by participants during the 7 days before PSG nights.

Daytime functioning was assessed by IDSIQ total score, a questionnaire completed every evening by the participants. IDSIQ comprises 14 questions grouped into three domains of sleepiness, alert/cognition and mood. The IDSIQ total score has a maximum of 140 points (each question being scored from 0 to 10 points) with lower scores indicating better daytime functioning. Please refer to Hudgens et al. (2021) for further details. The IDSIQ total and domain scores were calculated from the mean of daily entries by participants during the 7 days before PSG nights at baseline, Months 1 and 3. A ≥ 17-point reduction in the IDSIQ total score is considered clinically meaningful (Hudgens et al. 2021; Phillips-Beyer et al. 2023).

### 2.4 | Safety Endpoints

Safety data were collected from the signing of the consent form up to 30 days after the end of the double-blind study period or until the date of enrolment in the extension trial. Adverse events (AEs) included somnolence and fatigue, based on FDA MedDRA queries (FDA US Food and Drug administration 2022), because both are typical symptoms of insomnia and OSA, and common AEs of hypnotics. An independent safety board adjudicated blinded AEs associated with narcolepsy-like symptoms or suicide or self-injury. In addition, daytime somnolence was assessed using the Epworth Sleepiness Scale (ESS). The ESS scores were obtained by patient self-assessment onsite on the morning of the second PSG night at baseline, Months 1 and 3. Scores range from 0 to 24 with scores of up to 10 points considered as normal and scores 16–24 considered as severe excessive daytime sleepiness (Johns 1991). Next-morning residual effects were assessed by morning sleepiness VAS collected daily and average over the 7 days before PSG nights. VAS values were reported by participants in placing a mark on a VAS scale (mm) ranging from 0 ‘very sleepy’ to 100 ‘not sleepy at all’.

### 2.5 | Statistical Analysis

Efficacy analyses of participants with mild OSA were based on the intention-to-treat population, defined as all participants with mild OSA randomised to a study treatment (Full Analysis Set). All available data were included in the analysis and the results are reported according to the assigned study treatment. Safety analyses of participants with mild OSA included all participants who received at least one dose of study treatment, and outcomes were reported according to the study treatment taken (Safety Analysis Set).

Descriptive statistics are reported as means and standard deviations (SDs), standard errors of the means (SE) or 95% confidence intervals (CI) for quantitative variables, and frequencies and percentages for qualitative data.

The change from baseline in participants with mild OSA at Months 1 and 3 in WASO, LPS, sTST and IDSIQ total score was analysed using a linear mixed-effects model for repeated measures. The model was adjusted for the baseline value of the relevant response variable (either WASO, LPS, sTST or IDSIQ total score), age group (< 65; ≥ 65 years), treatment (daridorexant 50 mg; daridorexant 25 mg; placebo), visit (Months 1 and 3), interaction of treatment by visit and baseline by visit. Values are reported as least-squares mean (LSM) with 95% CI for each treatment group per time point. For each daridorexant dose comparison with placebo, the placebo-corrected LSM is reported together with the associated 95% CI and the unadjusted two-sided *p*-value. Two-sided *p*-values of < 0.05 were considered statistically significant. The subgroup analyses are exploratory, and no adjustments for multiplicity were made. Hence, the 95% CIs and *p*-values should be interpreted with caution.

Results of analyses performed on the counterpart subgroup of participants without OSA and on the overall population are reported in the Supporting Information. We concluded that there was no evidence of deviation from the results in the overall study population if the LSM of the overall population fell within the CI of the subgroup with mild OSA in the Forest plot.

Statistical analyses were performed using SAS software (version 9.4; SAS Institute, Cary, NC. USA).

## 3 | Results

### 3.1 | Mild-OSA Subgroup Population

Of the 930 participants with insomnia disorder randomised in the original Phase 3 study, 5 (< 1%) participants had missing AHI data at screening. Out of the remaining 925 participants, 153 (16.5%) were identified as having insomnia disorder and untreated mild OSA (AHI 5–< 15 events/h) at screening. The participants were equally distributed between the treatment arms, with 53, 53 and 47 receiving daridorexant 25 mg, daridorexant 50 mg, and placebo, respectively (Table 1). Their mean (SD) AHI at screening was 8.1 (2.5) events/h, ranging from 7.7 (2.2) events/h to 8.4 (2.9) events/h across the treatment arms (Table 1).

In this subgroup with mild OSA, baseline demographics of age, body-mass index (BMI) and sex were similar across the treatment arms (Table 1). Compared with the overall study population, participants were older (mean age 63.8 vs. 55.4 years; with 61.4% vs. 39.1% of participants being ≥ 65 years old), had a higher mean BMI (28.4 vs. 26.4 kg/m2) and were more likely to be male (39.9% vs. 32.9%) (Tables 1 and S1). Other baseline demographics, such as race and ethnicity, were similar to the overall study population.

Baseline insomnia characteristics including ISI total score, WASO, LPS, sTST, IDSIQ total score, VAS morning sleepiness and ESS were well balanced between the treatment arms (Table 1). The mean ISI total score was 18.5 (ranging from 18.3 to 18.7), mean LPS was 64.9 min (ranging from 58.3 to 69.7 min), mean WASO was 114.2 min (ranging from 107.8 to 128.0 min), mean sTST was 311.0 min (ranging from 302.0 to 324.2 min) and mean IDSIQ total score was 66.4 (ranging from 65.5 to 67.9). Of note, baseline insomnia characteristics were similar to the overall study population with the exception of WASO, which was longer in participants with mild OSA (mean: 114.2 vs. 98.6 min) (Tables 1 and S1).

### 3.2 | Night-Time Efficacy Endpoints in Participants With Mild OSA

**FIGURE 1 |:**
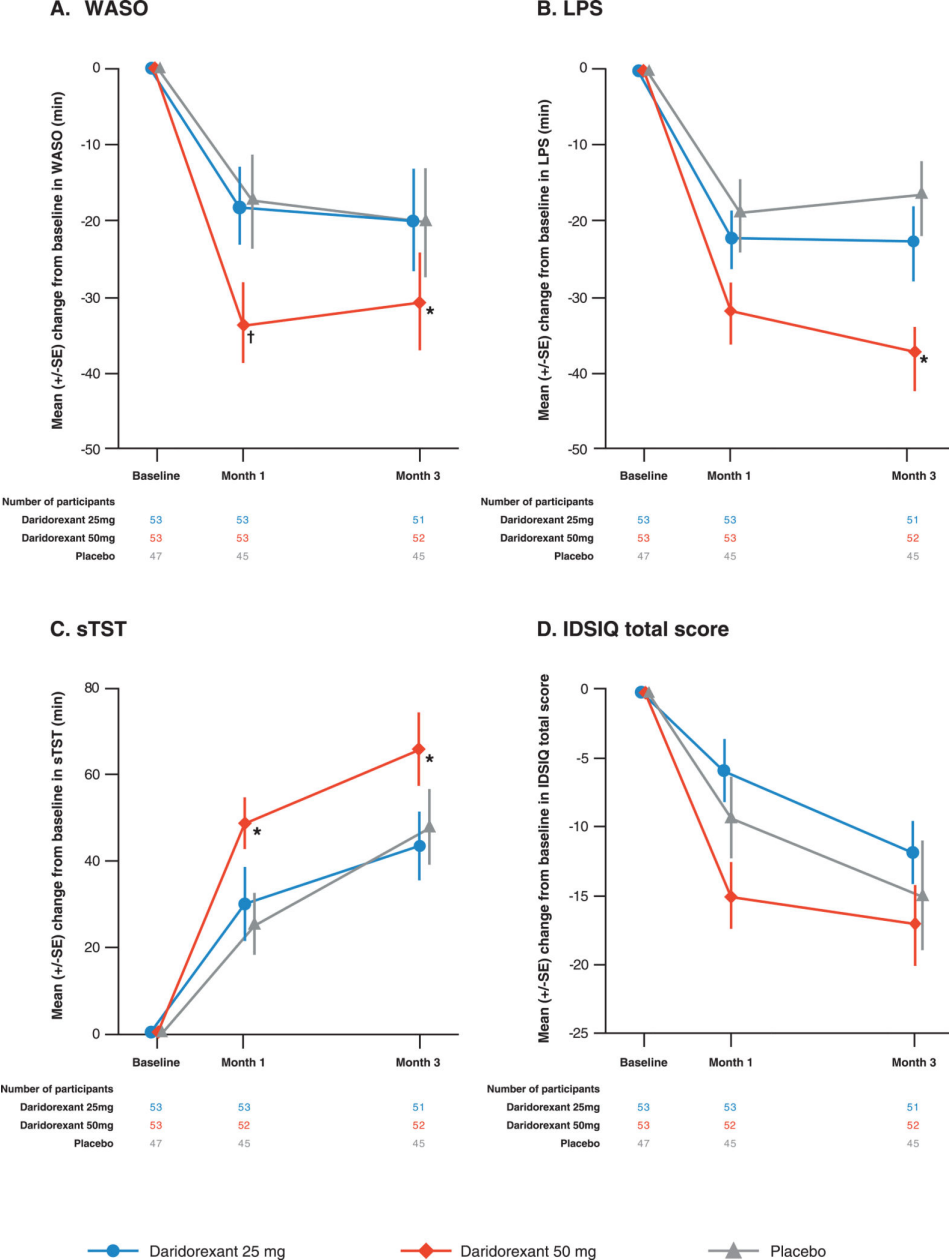
Efficacy of daridorexant on night-time and daytime parameters in participants with comorbid insomnia disorder and mild OSA. Mean change from baseline of observed WASO (A), LPS (B), sTST (C) and IDSIQ total score (D) at Months 1 and 3. Error bars show SE. Two-sided *p*-values for the difference to placebo of the adjusted least squares means are included for each figure panel. IDSIQ, Insomnia Daytime Symptoms and Impacts Questionnaire; LPS, latency to persistent sleep; sTST, self-reported total sleep time; WASO, wake time after sleep onset; SE, standard errors. *p < 0.05; †*p* < 0.001.

Daridorexant 50 mg improved WASO from baseline to Months 1 and 3 by −37.7 min (LSM; 95% CI −47.0 to −28.4) and −35.4 min (95% CI −46.6 to −24.1), respectively (Figure 1 and Table S2). Differences to placebo were significant at both timepoints (Month 1: −24.0 min, *p* = 0.0009; Month 3: −19.8 min, *p* = 0.0203) (Figure 1 and Table S2). Although daridorexant 25 mg improved WASO from baseline to Month 1 (−23.7 min [95% CI −33.1 to −14.3]) and Month 3 (−25.2 min [95% CI −36.5 to −13.8]), differences to placebo were not significant at either timepoint (Month 1: −10.0 min, *p* = 0.1591; Month 3: −9.7 min, *p* = 0.2552) (Figure 1 and Table S2).

Daridorexant 50 mg improved LPS from baseline to Months 1 and 3 by −31.0 min (LSM; 95% CI −38.2 to −23.8) and −36.9 min (95% CI −45.6 to −28.3), respectively (Figure 1and Table S2). The difference to placebo was significant at Month 3 (Month 1: −10.0 min, *p* = 0.0668; Month 3: −18.9, *p* = 0.0039). Although daridorexant 25 mg improved LPS at Month 1 (−18.9 min [95% CI −26.2 to −11.7]) and Month 3 (−20.8 min [95% CI −29.6 to −12.0]), differences to placebo were not significant at either timepoint (Figure 1 and Table S2).

Daridorexant 50 mg improved sTST from baseline to Months 1 and 3 by LSM 49.9 min (95% CI 36.0–63.8) and 69.4 min (95% CI 53.7–85.1), respectively (Figure 1 and Table S2). Differences to placebo were significant at both timepoints (Month 1: 26.3 min, *p* = 0.0119; Month 3: 24.6 min, *p* = 0.0370) (Figure 1 and Table S2). The differences to placebo in mean sTST with daridorexant 25 mg were not significant at Month 1 (4.8 min; *p* = 0.6410) or Month 3 (−0.0 min; *p* = 0.9995) (Figure 1 and Table S2).

No marked deviations from the results of the overall population and the subgroup without OSA were observed in the results on night-time sleep parameters in participants with mild OSA (Figure S1, Tables S2 and S3).

### 3.3 | Daytime Efficacy Endpoints in Participants With Mild OSA

Daridorexant 50 mg improved the mean IDSIQ total score from baseline to Month 1 and Month 3 by LSM −14.4 (95% CI −19.1 to −9.6) and −17.2 (95% CI −23.2 to −11.1), respectively (Figure 1 and Table S2). Differences to placebo were not significant at either timepoint (Month 1: −5.2, *p* = 0.1393; Month 3: −2.2, *p* = 0.6201) (Figure 1 and Table S2). Daridorexant 25 mg improved the mean IDSIQ total score from baseline to Months 1 and 3 by −6.0 (95% CI −10.8 to −1.3) and −11.6 (95% CI −17.7 to −5.5), respectively. The differences to placebo in mean IDSIQ total score with daridorexant 25 mg were not significant at Month 1 (+3.1; *p* = 0.3760) or Month 3 (+3.3; *p* = 0.4655) (Figure 1 and Table S2). The effect of daridorexant 50 and 25 mg on the IDSIQ domain scores for sleepiness, mood and alert/cognition followed a similar pattern as the IDSIQ total score without reaching significant differences to placebo with either dose (Figures S2 and S3).

### 3.4 | Safety Analysis in Participants With Mild OSA

The prevalence of AEs (Table 2) was 41.5% (daridorexant 50 mg; *N* = 53), 28.2% (daridorexant 25 mg; *N* = 53) and 31.9% (placebo; *N* = 47). The most common AEs that occurred in at least 3% of participants in any treatment arm during the double-blind treatment period (Table 2) included somnolence (*n* = 1 [1.9%], *n* = 5 [9.4%] and none in participants receiving daridorexant 50, 25 mg, and placebo, respectively), headache (*n* = 2 [3.8%], *n* = 3 [5.7%] and *n* = 1 [2.1%] in participants receiving daridorexant 50, 25 mg and placebo, respectively), and falls (*n* = 2 [4.3%] in the placebo arm, none with daridorexant 25 or 50 mg) (Table 2). Two participants in the daridorexant 50 mg treatment arm had serious AEs that were considered unrelated to the study treatment (Table 2). None of the participants receiving daridorexant discontinued study treatment due to AEs in contrast to two (4.3%) participants receiving placebo. Independent safety board-adjudicated AEs were reported for participants receiving daridorexant 50 mg: two participants had excessive daytime sleepiness, and three participants had sleep paralysis. No participants reported hallucinations or suicidal ideation/self-injury.

No worsening of next-morning sleepiness measured by VAS morning sleepiness was observed in any treatment group at Months 1 or 3 (Table 3). In fact, a trend towards improvement was observed in all treatment arms with numerically greater improvement with daridorexant 50 mg compared with placebo at Months 1 and 3. Daridorexant 50 mg improved VAS morning sleepiness by 9.7 mm from baseline to Month 1 and by 15.1 mm to Month 3. Daridorexant 25 mg improved VAS morning sleepiness by 6.9 mm from baseline to Month 1 and by 10.5 mm to Month 3. Improvements in the placebo group were 4.9 at Month 1 and 12.1 at Month 3 (Table 3).

Daytime-sleepiness measured by ESS scores ranged from 6.0 to 6.8 across the treatment arms. A slight numerical improvement was observed across all treatment arms over time (changes from baseline to Month 3 ranged from −1.1 to −1.6 (Table S4)).

The safety profile of participants with mild OSA was similar to that of participants without OSA (Tables 2 and S5), and the overall Phase 3 study population (Mignot et al. 2022).

## 4 | Discussion

This post hoc subgroup analysis is the first assessment of daridorexant on insomnia symptoms in participants with comorbid insomnia disorder and untreated mild OSA. It showed that daridorexant 50 mg was well tolerated and improved insomnia sleep parameters in these patients.

The baseline characteristics of the ISI total score, WASO, LPS, sTST and IDSIQ total score show that participants with mild OSA had moderate-to-severe insomnia symptoms with pronounced difficulty to initiate and maintain sleep. Of note, baseline insomnia characteristics were generally similar to the overall Phase 3 study population, with the exception of WASO, which was longer in participants with mild OSA. This observation is consistent with previous reports (Huttunen et al. 2021). Participants with mild OSA also had a higher BMI, were older and were more likely to be male compared with the overall Phase 3 study population. This finding is in line with expectations, as a higher BMI, advanced age and male sex are risk factors for OSA (Senaratna et al. 2017). Therefore, the characteristics of this subgroup are in line with real-world observations of patients with comorbid insomnia disorder and OSA (Huttunen et al. 2021).

Our analysis shows that daridorexant 50 mg improved sleep parameters (WASO, LPS and sTST) in patients with comorbid insomnia disorder and mild OSA. Daridorexant 50 mg also showed a trend to improved daytime functioning as assessed by the IDSIQ total score and the three IDSIQ domain scores, although the differences to placebo with both daridorexant doses in IDSIQ total score were not significant. At Month 3, daridorexant 50 mg reached the ≥ 17-point reduction in IDSIQ total score that is considered clinically meaningful (Hudgens et al. 2021; Phillips-Beyer et al. 2023). Importantly, the efficacy outcomes in participants with mild OSA did not deviate from results observed in the overall study population.

No safety concerns were observed with either dose of daridorexant versus placebo during the 3 months of the study in patients with comorbid insomnia disorder and mild OSA. The prevalences of AEs were low and no evidence of dose dependency with daridorexant was observed, including daytime somnolence and fatigue (Mignot et al. 2022; Luyet et al. 2024). These findings indicate that daridorexant is well tolerated in participants with mild OSA. Daytime somnolence is of special interest, as it is a typical symptom of both OSA and insomnia disorder affecting the quality of life of patients (Gottlieb and Punjabi 2020; Riemann et al. 2022). In addition, VAS morning sleepiness scores showed that there was no residual effect of daridorexant 25 or 50 mg on next-morning sleepiness. In fact, a trend to reduced next-morning sleepiness was observed in all three treatment arms, with daridorexant 50 mg having the most pronounced effect. Furthermore, daridorexant (25 or 50 mg) did not increase daytime sleepiness as assessed by ESS. ESS scores at baseline were within the normal range in all three treatment arms and there was therefore limited room to detect any improvement (Treiber et al. 2017).

In clinical practice, questions remain regarding how to treat patients with insomnia disorder and mild OSA. Existing guidelines do not provide specific recommendations for these patients. One objective for pharmacological interventions in COMISA is the treatment of insomnia symptoms without worsening OSA. The effects of hypnotics on sleep and nighttime respiration vary between patients with OSA (Mason et al. 2015; Carter and Eckert 2021). Many sleep medications, such as benzodiazepines, may impair oxygen saturation and, therefore, require cautionary use in respiratory-depressed patients (Riemann et al. 2023; Mason et al. 2015; Carter and Eckert 2021). Another concern of hypnotic use is their potential exacerbation of next-day symptoms such as daytime somnolence (Mason et al. 2015; Carter and Eckert 2021). Also, some hypnotics including benzodiazepines, benzodiazepine receptor agonists and low-dose sedating antidepressants are indicated primarily for short-term treatment of insomnia disorder (≤ 4 weeks) due to safety concerns, as longer-term use can lead to development of tolerance and dependence, nocturnal confusion and falls (Riemann et al. 2023; Carter and Eckert 2021). Although OSA disease characteristics were not assessed throughout the Phase 3 study of daridorexant, two Phase 1 studies in patients with mild and moderate OSA (AHI 5–< 30 events/h; NCT03765294) and in patients with severe OSA (AHI ≥ 30 events/h; NCT05458193) showed that daridorexant 50 mg did not impair night-time respiratory function and improved sleep parameters independent of OSA severity (Boof et al. 2021, 2023). Of note, participants in the Phase 1 studies did not have concomitant insomnia disorder, and the small sample sizes (*n* = 28 and *n* = 16, respectively) and short study period (5 days) prevent the extrapolation of the results to long-term treatment in COMISA (Boof et al. 2021, 2023). Our data from this subgroup analysis indicate that daridorexant was well tolerated and offers an effective option to treat insomnia symptoms without worsening next-day functioning in patients with comorbid insomnia disorder and mild OSA.

This subgroup analysis has several limitations. By its post hoc nature, *p*-values were descriptive, and results should be confirmed in an independent study. The treatment arms of the mild OSA subgroup have small sample sizes (*n* = 53, *n* = 53, *n* = 47 for daridorexant 50, 25 mg and placebo, respectively), resulting in large confidence intervals for efficacy outcomes. In addition, participants in the Phase 3 study were selected based on their insomnia symptoms. Mild OSA was identified as a patient characteristic during screening and was untreated. Furthermore, OSA criteria were not considered in the randomisation. Therefore, results cannot be generalised to patients with comorbid insomnia disorder and OSA across the severity spectrum. In addition, it was not possible to assess the effect of daridorexant on night-time respiratory function, because AHI data and oxygen saturation were not collected during the treatment period. Although the effect of daridorexant on OSA disease characteristics was not formally assessed, the outcomes on next-morning sleepiness and ESS indicate that daridorexant does not worsen next-day daytime sleepiness.

The strengths of this analysis assessing daridorexant in patients with comorbid insomnia disorder and mild OSA include the robust source of data from a prospective clinical trial evaluating night-time and daytime sleep measures. In these patients, the analysis assessed a dual orexin receptor antagonist with established efficacy and safety in patients with insomnia disorder. Moreover, as outlined above, the mild OSA subgroup of this analysis is representative of patients in the real world based on BMI, age, proportion of male gender in the subgroup and the proportion of patients with mild OSA within the overall study population.

## 5 | Conclusion

In conclusion, in participants with comorbid insomnia disorder and untreated mild OSA, daridorexant 50 mg improved sleep parameters without increasing daytime sleepiness and had a safety profile comparable to that of placebo. These results indicate that daridorexant 50 mg may provide a promising approach to improve the management of these patients. Further research is needed to investigate the impact of daridorexant on parameters of OSA, such as AHI and hypoxic burden, and its efficacy and safety on sleep and daytime functioning in individuals with comorbid insomnia and OSA across the severity spectrum.

## Supplementary Material

sup

Supporting Information

Additional supporting information can be found online in the Supporting Information section.

## Figures and Tables

**TABLE 1 |:** Demographics and baseline characteristics in participants with comorbid insomnia disorder and mild OSA (AHI: 5–< 15 events/h).

	Daridorexant 50 mg	Daridorexant 25 mg	Placebo	Total
	*N* = 53	*N* = 53	*N* = 47	*N* = 153
*Full analysis set*				
Age at screening, years			
Mean (SD)	63.6 (12.5)	63.6 (10.4)	64.3 (11.3)	63.8 (11.4)
< 65 [*n* (%)]	23 (43.4)	20 (37.7)	16 (34.0)	59 (38.6)
≥ 65 [*n* (%)]	30 (56.6)	33 (62.3)	31 (66.0)	94 (61.4)
Sex, *n* (%)				
Male	21 (39.6)	19 (35.8)	21 (44.7)	61 (39.9)
Race, *n* (%)				
Asian	1 (1.9)	0	2 (4.3)	3 (2.0)
Black or African American	3 (5.7)	3 (5.7)	3 (6.4)	9 (5.9)
White	49 (92.5)	50 (94.3)	42 (89.4)	141 (92.2)
Other	0	0	0	0
Ethnicity, *n* (%)				
Hispanic or Latino	7 (13.2)	11 (20.8)	7 (14.9)	25 (16.3)
Not Hispanic or Latino	45 (84.9)	42 (79.2)	40 (85.1)	127 (83.0)
Unknown	1 (1.9)	0	0	1 (0.7)
BMI, kg/m2				
Mean (SD)	27.9 (4.5)	28.9 (4.0)	28.3 (4.4)	28.4 (4.3)
< 25 [*n* (%)]	16 (30.2)	7 (13.2)	11 (23.4)	34 (22.2)
25–≤ 30 [*n* (%)]	23 (43.4)	28 (52.8)	22 (46.8)	73 (47.7)
> 30 [*n* (%)]	14 (26.4)	18 (34.0)	14 (29.8)	46 (30.1)
ISI total score, score				
Mean (SD)	18.5 (3.9)	18.3 (5.2)	18.7 (4.0)	18.5 (4.4)
PSG WASO, min				
Mean (SD)	108.4 (43.7)	107.8 (33.1)	128.0 (42.7)	114.2 (40.8)
PSG LPS, min				
Mean (SD)	66.1 (31.6)	69.7 (43.5)	58.3 (33.9)	64.9 (36.8)
sTST, min				
Mean (SD)	324.2 (56.9)	305.7 (79.2)	302.0 (61.5)	311.0 (67.0)
IDSIQ total score				
Mean (SD)	67.9 (22.3)	65.5 (24.5)	65.7 (22.3)	66.4 (23.0)
AHI at screening, score				
Mean (SD)	8.4 (2.9)	7.7 (2.2)	8.2 (2.5)	8.1 (2.5)
*Safety analysis set* VAS morning sleepiness, mm				
Mean (SD)	41.1 (18.8)	42.4 (20.2)	45.3 (21.2)	42.8 (20.0)
ESS total score				
Mean (SD)	6.8 (5.4)	6.3 (5.2)	6.0 (5.1)	6.4 (5.2)

Abbreviations: AHI, apnoea-hypopnoea index; BMI, body-mass index; ESS, Epworth Sleepiness Scale; IDSIQ, Insomnia Daytime Symptoms and Impacts Questionnaire; ISI, insomnia severity index; LPS, latency to persistent sleep; OSA, obstructive sleep apnoea; PSG, polysomnography; SD, standard deviation; sTST, self-reported total sleep time; VAS, visual analogue scale; WASO, wake time after sleep onset.

**TABLE 2 |:** Adverse events in participants with comorbid insomnia disorder and mild OSA.

	Daridorexant 50 mg (*n* = 53)	Daridorexant 25 mg (*n* = 53)	Placebo (*n* = 47)
Participants with ≥ 1 AE^a^	22 (41.5)	15 (28.3)	15 (31.9)
AEs^a^ of ≥ 3% in any group (preferred term) Somnolence	1 (1.9)	5 (9.4)	0
Headache	2 (3.8)	3 (5.7)	1 (2.1)
Fall	0	0	2 (4.3)
Accidental overdose	2 (3.8)	2 (3.8)	0
Haemoglobin decrease	2 (3.8)	0	0
Nausea	2 (3.8)	0	0
Pruritus	2 (3.8)	0	0
AEs^a^ leading to treatment discontinuation	0	0	2 (4.3)
Participants with ≥ 1 SAE^b^	2 (3.8)	0	0
Related to study treatment	0	0	0
Adjudicated AEs^c^ Excessive daytime sleepiness	2 (3.8)	0	0
Sleep paralysis	3 (5.7)	0	0
Hallucinations	0	0	0
Suicidal ideation or self-injury	0	0	0

*Note:* Data are *n* (%).

Abbreviations: AE, adverse event; OSA, obstructive sleep apnoea; SAE, serious adverse event; TEAE, treatment-emergent adverse event.

a Includes AEs that occurred during the double-blind treatment period with their preferred terms.

b SAEs in two participants receiving daridorexant 50 mg included one participant with syncope, post-procedural haemorrhage and decreased haemoglobin and one participant with renal colic. SAEs were reported during the double-blind study period up to 30 days after the end of treatment (or date of enrolment into the extension study).

c Adjudicated AEs were reported during the double-blind study period up to 30 days after the end of treatment (or date of enrolment into the extension study) and were adjudicated blindly by an independent safety board.

**TABLE 3 |:** Next-morning residual effect measured by VAS morning sleepiness in participants with comorbid insomnia disorder and mild OSA.

	Daridorexant 50 mg (*n* = 53)	Daridorexant 25 mg (*n* = 53)	Placebo (*n* = 47)
VAS morning sleepiness at baseline (mm)			
*n*	53	53	47
Mean (SD)	41.1 (18.8)	42.4 (20.2)	45.3 (21.2)
Change from baseline at Month 1 (mm)			
*n*	52	53	45
Mean (SD)	9.7 (16.9)	6.9 (15.4)	4.9 (17.1)
Change from baseline at Month 3 (mm)			
*n*	52	51	45
Mean (SD)	15.1 (22.1)	10.5 (18.1)	12.1 (18.8)

Abbreviations: OSA, obstructive sleep apnoea; SD, standard deviation; VAS, visual analogue scale.

## Data Availability

In addition to Idorsia’s existing clinical trial disclosure activities, the company is committed to implementing the Principles for Responsible Clinical Trial Data Sharing jointly issued by the European Federation of Pharmaceutical Industries and Associations (EFPIA) and the Pharmaceutical Research and Manufacturers of America (PhRMA). Requests for data sharing, of any level, can be directed to clinical-trials-disclosure@idorsia.com for medical and scientific evaluation.

## Abbreviations:

AHI: apnoea-hypopnoea index
BMI: body-mass index
CBT: cognitive-behavioural therapy
CI: confidence interval
COMISA: untreated mild OSA and comorbid insomnia disorder
ESS: Epworth Sleepiness Scale
IDSIQ: Insomnia Daytime Symptoms and Impacts Questionnaire
ISI: insomnia severity index
LPS: latency to persistent sleep
LSM: least-squares mean
OSA: obstructive sleep apnoea
PSG: polysomnography
SD: standard deviation
SE: standard error
sTST: self-reported total sleep time
VAS: visual analogue scale
WASO: wake after sleep onset
