# Supplementary material for: The Effects of Daridorexant on Patients With Comorbid Insomnia Disorder and Untreated Mild Obstructive Sleep Apnoea: A Post Hoc Subgroup Analysis of a Phase 3, Randomised Clinical Trial
Source: J Sleep Res. Author manuscript; Available in PMC 2026 Feb 25. (PMC12931678; doi:10.1111/jsr.70135)
Supplement: Supplementary file 1 [file NIHMS2097756-supplement-sup.docx]

# **SUPPLEMENTARY MATERIALS**

## **List of captions for supplementary tables and figures**

**Table S1**: Baseline demographics and characteristics in participants with insomnia disorder without OSA and the overall phase 3 study population

**Table S2**: Change from baseline in night-time and daytime efficacy measures in participants with comorbid insomnia disorder and mild OSA

**Table S3**: Change from baseline in night-time and daytime efficacy measures in participants with insomnia disorder without OSA

**Figure S1**: Efficacy of daridorexant on night-time (WASO, LPS, sTST) and daytime (IDSIQ total score) parameters in participants with insomnia disorder and mild OSA, without OSA, and in the overall phase 3 study population

**Figure S2:** Efficacy of daridorexant on IDSIQ domain scores in participants with comorbid insomnia disorder and mild OSA 

**Figure S3**: Efficacy of daridorexant on IDSIQ domain scores in participants with insomnia disorder and mild OSA, without OSA, and in the overall phase 3 study population

**Table S4**: Subjective daytime sleepiness measured by Epworth Sleepiness Scale© in participants with comorbid insomnia disorder and mild OSA

**Table S5**: Adverse events in the safety analysis set of participants without OSA (n=270)

## **Table S1****: Baseline demographics and characteristics in participants with insomnia disorder without OSA (AHI <5 events/hour) and the overall phase 3 study population**

|  | **Overall phase 3 randomized participants** | **Subgroup without OSA** **AHI < 5 events/hour** |
| --- | --- | --- |
| **Full Analysis Set** | **n=930** | **n=772** |
| **Age at screening, years** |  |  |
| Mean (SD) | 55.4 (15.3) | 53.7 (15.5) |
| < 65 [n (%)] | 566 (60.9) | 504 (65.3) |
| ≥65 [n (%)] | 364 (39.1) | 268 (34.7) |
| **Sex, n (%)** |  |  |
| Male | 306 (32.9) | 244 (31.6) |
| **Race, n (%)** |  |  |
| Asian | 9 (1.0) | 6 (0.8) |
| Black or African American | 77 (8.3) | 68 (8.8) |
| White | 839 (90.2) | 693 (89.8) |
| Other | 5 (0.5) | 5 (0.6) |
| **Ethnicity, n (%)** |  |  |
| Hispanic or Latino | 146 (15.7) | 121 (15.7) |
| Not Hispanic or Latino | 783 (84.2) | 651 (84.3) |
| Unknown | 1 (0.1) | 0 |
| **BMI, kg/m**^**2**^ |  |  |
| Mean (SD) | 26.4 (4.3) | 26.1 (4.1) |
| < 25 [n (%)] | 367 (39.5) | 331 (42.9) |
| 25–≤30 [n (%)] | 388 (41.7) | 312 (40.4) |
| >30 [n (%)] | 175 (18.8) | 129 (16.7) |
| **ISI**© **total Score, score** |  |  |
| Mean (SD) | 19.1 (4.1) | 19.2 (4.1) |
| **PSG WASO, min** |  |  |
| Mean (SD) | 98.6 (39.2) | 95.5 (38.1) |
| **PSG LPS, min** |  |  |
| Mean (SD) | 65.8 (38.6) | 65.8 (38.7) |
| **sTST, min** |  |  |
| Mean (SD) | 313.0 (57.0) | 313.4 (54.8) |
| **IDSIQ total score** |  |  |
| Mean (SD) | 73.7 (24.8) | 75.1 (24.9) |
| **AHI at screening, score** |  |  |
| Mean (SD) | 2.4 (3.0) | 1.2 (1.3) |
| **Safety Analysis Set** | **n=927** | **n=770** |
| **VAS morning sleepiness, mm** |  |  |
| Mean (SD) | 37.5 (18.8) | 36.5 (18.4) |
| **ESS total score** |  |  |
| Mean | 6.1 (5.4) | 6.0 (5.4) |

AHI, apnea-hypopnea index; BMI, body-mass index; ESS©, Epworth Sleepiness Scale; IDSIQ, Insomnia Daytime Symptoms and Impacts Questionnaire; ISI©, insomnia severity index; LPS, latency to persistent sleep; OSA, obstructive sleep apnea; PSG, polysomnography; SD, standard deviation; sTST, self-reported total sleep time; VAS, Visual Analog Scale; WASO, wake time after sleep onset.

## **Table S2: Change from baseline in night-time and daytime efficacy measures in participants with comorbid insomnia disorder and mild OSA**

|  | **Month 1** |  |  | **Month 3** |  |  |
| --- | --- | --- | --- | --- | --- | --- |
|  | **Daridorexant** **50 mg** **(n=53)** | **Daridorexant** **25 mg** **(n=53)** | **Placebo** **(n=47)** | **Daridorexant** **50 mg** **(n=53)** | **Daridorexant** **25 mg** **(n=53)** | **Placebo** **(n=47)** |
| **WASO (min)** |  |  |  |  |  |  |
| Baseline, mean (SD) | 108.4 (43.7) | 107.8 (33.1) | 128.0 (42.7) | 108.4 (43.7) | 107.8 (33.1) | 128.0 (42.7) |
| Change from baseline,  LS mean (95% CI) | -37.7 (-47.0, -28.4) | -23.7 (-33.1, -14.3) | -13.8 (-24.1, -3.5) | -35.4 (-46.6, -24.1) | -25.2 (-36.5, -13.8) | -15.5 (-27.8, -3.3) |
| Difference to Placebo,  LS mean (95% CI) | -24.0 (-37.9, -10.0) | -10.0 (-23.9, 3.9) | - | -19.8 (-36.5, -3.1) | -9.7 (-26.4, 7.0) | - |
| Two-sided p-value | 0.0009 | 0.1591 | - | 0.0203 | 0.2552 | - |
| **LPS (min)** |  |  |  |  |  |  |
| Baseline, mean (SD) | 66.1 (31.6) | 69.7 (43.5) | 58.3 (33.9) | 66.1 (31.6) | 69.7 (43.5) | 58.3 (33.9) |
| Change from baseline,  LS mean (95% CI) | -31.0 (-38.2, -23.8) | -18.9 (-26.2, -11.7) | -21.0 (-28.9, -13.1) | -36.9 (-45.6, -28.3) | -20.8 (-29.6, -12.0) | -18.1 (-27.4, -8.7) |
| Difference to Placebo,  LS mean (95% CI) | -10.0 (-20.6, 0.7) | 2.1 (-8.6, 12.8) | - | -18.9 (-31.6, -6.2) | -2.8 (-15.5, 10.0) | - |
| Two-sided p-value | 0.0668 | 0.7038 | - | 0.0039 | 0.6715 | - |
| **sTST (min)** |  |  |  |  |  |  |
| Baseline, mean (SD) | 324.2 (56.9) | 305.7 (79.2) | 302.0 (61.5) | 324.2 (56.9) | 305.7 (79.2) | 302.0 (61.5) |
| Change from baseline,  LS mean (95% CI) | 49.9 (36.0, 63.8) | 28.4 (14.5, 42.2) | 23.6 (8.6, 38.6) | 69.4 (53.7, 85.1) | 44.9 (29.1, 60.6) | 44.9 (28.0, 61.8) |
| Difference to Placebo,  LS mean (95% CI) | 26.3 (5.9, 46.7) | 4.8 (-15.4, 24.9) | - | 24.6 (1.5, 47.6) | -0.0 (-22.9, 22.9) | - |
| Two-sided p-value | 0.0119 | 0.6410 | - | 0.0370 | 0.9995 | - |
| **IDSIQ total score** |  |  |  |  |  |  |
| Baseline, mean (SD) | 67.9 (22.3) | 65.5 (24.5) | 65.7 (22.3) | 67.9 (22.3) | 65.5 (24.5) | 65.7 (22.3) |
| Change from baseline,  LS mean (95% CI) | -14.4 (-19.1, -9.6) | -6.0 (-10.8, -1.3) | -9.1 (-14.3, -4.0) | -17.2 (-23.2, -11.1) | -11.6 (-17.7, -5.5) | -14.9 (-21.5, -8.3) |
| Difference to Placebo,  LS mean (95% CI) | -5.2 (-12.2, 1.7) | 3.1 (-3.8, 10.0) | - | -2.2 (-11.1, 6.7) | 3.3 (-5.6, 12.2) | - |
| Two-sided p-value | 0.1393 | 0.3760 | - | 0.6201 | 0.4655 | - |

Baseline mean is the average of the observed values and the LS mean is the adjusted mean for the baseline value of the relevant response variable, age group (< 65 years; ≥ 65 years), treatment (daridorexant 50 mg; daridorexant 25 mg; placebo), visit (Month 1; Month 3), interaction of treatment by visit, and baseline by visit. 

CI, confidence interval; IDSIQ, Insomnia Daytime Symptoms and Impacts Questionnaire; LPS, latency to persistent sleep; LSM, least squares mean; OSA, obstructive sleep apnea; sTST, self-reported total sleep time; WASO, wake time after sleep onset.

## **Table S3: Change from baseline in night-time and daytime efficacy measures in participants with insomnia disorder without OSA**

|  | **Month 1** |  |  | **Month 3** |  |  |
| --- | --- | --- | --- | --- | --- | --- |
|  | **Daridorexant**  **50 mg** **(n=256)** | **Daridorexant** **25 mg** **(n=255)** | **Placebo** **(n=261)** | **Daridorexant**  **50 mg** **(n=256)** | **Daridorexant** **25 mg** **(n=255)** | **Placebo** **(n=261)** |
| **WASO (min)** |  |  |  |  |  |  |
| Baseline, mean (SD) | 92.8 (36.0) | 95.6 (39.5) | 98.0 (38.8) | 92.8 (36.0) | 95.6 (39.5) | 98.0 (38.8) |
| Change from baseline,  LS mean (95% CI) | -27.1 (-31.2, -23.1) | -17.2 (-21.3, -13.1) | -4.5 (-8.5, -0.4) | -28.2 (-32.5, -24.0) | -22.4 (-26.6, -18.1) | -9.8 (-14.1, -5.6) |
| Difference to Placebo,  LS mean (95% CI) | -22.6 (-28.3, -17.0) | -12.7 (-18.4, -7.0) | - | -18.4 (-24.3, -12.5) | -12.5 (-18.4, -6.6) | - |
| Two-sided p-value | <0.0001 | <0.0001 | - | <0.0001 | <0.0001 | - |
| **LPS (min)** |  |  |  |  |  |  |
| Baseline, mean (SD) | 63.1 (38.5) | 66.2 (36.8) | 68.2 (40.6) | 63.1 (38.5) | 66.2 (36.8) | 68.2 (40.6) |
| Change from baseline,  LS mean (95% CI) | -31.1 (-34.8, -27.4) | -30.1 (-33.8, -26.3) | -18.9 (-22.5, -15.2) | -34.3 (-37.8, -30.8) | -32.9 (-36.4, -29.4) | -23.3 (-26.7, -19.8) |
| Difference to Placebo,  LS mean (95% CI) | -12.3 (-17.4, -7.1) | -11.2 (-16.4, -6.0) | - | -11.0 (-15.9, -6.1) | -9.6 (-14.5, -4.7) | - |
| Two-sided p-value | <0.0001 | <0.0001 | - | <0.0001 | 0.0001 | - |
| **sTST (min)** |  |  |  |  |  |  |
| Baseline, mean (SD) | 310.9 (57.6) | 310.7 (55.4) | 318.4 (51.3) | 310.9 (57.6) | 310.7 (55.4) | 318.4 (51.3) |
| Change from baseline,  LS mean (95% CI) | 42.4 (36.4, 48.3) | 35.0 (29.0, 41.1) | 20.4 (14.5, 26.3) | 55.3 (48.1, 62.5) | 48.0 (40.8, 55.2) | 35.6 (28.5, 42.8) |
| Difference to Placebo,  LS mean (95% CI) | 22.0 (13.7, 30.2) | 14.7 (6.4, 23.0) | - | 19.6 (9.6, 29.7) | 12.4 (2.4, 22.4) | - |
| Two-sided p-value | <0.0001 | 0.0006 | - | 0.0001 | 0.0155 | - |
| **IDSIQ total score** |  |  |  |  |  |  |
| Baseline, mean (SD) | 75.9 (25.5) | 74.5 (24.4) | 74.9 (24.9) | 75.9 (25.5) | 74.5 (24.4) | 74.9 (24.9) |
| Change from baseline,  LS mean (95% CI) | -13.2 (-15.2, -11.3) | -9.7 (-11.7, -7.7) | -5.7 (-7.6, -3.7) | -19.7 (-22.3, -17.1) | -16.2 (-18.8, -13.6) | -11.6 (-14.1, -9.0) |
| Difference to Placebo,  LS mean (95% CI) | -7.5 (-10.3, -4.8) | -4.5 (-6.8, -1.3) | - | -8.2 (-11.8, -4.6) | -4.6 (-8.3, -1.0) | - |
| Two-sided p-value | <0.0001 | 0.0039 | - | <0.0001 | 0.0122 | - |

Baseline mean is the average of the observed values and the LS mean is the adjusted mean for the baseline value of the relevant response variable, age group (< 65 years; ≥ 65 years), treatment (daridorexant 50 mg; daridorexant 25 mg; placebo), visit (Month 1; Month 3), interaction of treatment by visit, and baseline by visit. 

CI, confidence interval; IDSIQ, Insomnia Daytime Symptoms and Impacts Questionnaire; LPS, latency to persistent sleep; LSM, least squares mean; OSA, obstructive sleep apnea; sTST, self-reported total sleep time; WASO, wake time after sleep onset. 

## **Figure S1: Efficacy of daridorexant on night-time and daytime parameters in participants with insomnia disorder and mild OSA, no OSA, and the overall phase 3 study population**  
Forest plots of the placebo-corrected LS mean (95% CI) of WASO (min) (A), LPS (min) (B), sTST (min) (C), and IDSIQ total score (D) of the change from baseline to Month 1 and Month 3 in participants receiving daridorexant 25 mg or 50 mg. LS means were adjusted for the baseline value of the relevant parameter (WASO, LPS, sTST or IDSIQ total score), age group (< 65 years; ≥ 65 years), treatment (daridorexant 50 mg; daridorexant 25 mg; placebo), visit (Month 1; Month 3), interaction of treatment by visit, and baseline by visit. Solid vertical line indicates the LS mean of the overall study population as reference; dashed vertical line is the effect threshold. 
CI, Confidence Interval; Fav, favours; LS mean, least square mean; OSA, obstructive sleep apnea; WASO, wake time after sleep onset.

## **Figure S2: Efficacy of daridorexant on IDSIQ domain scores in participants with comorbid insomnia disorder and mild OSA** 

Mean change from baseline of observed IDSIQ sleepiness domain (A), IDSIQ mood domain (B), and IDSIQ alert/cognition domain (C) at Month 1 and Month 3 in participants receiving daridorexant 50 mg, daridorexant 25 mg or placebo. Error bars show SE. Two-sided p-values for the difference to placebo indicated no significant differences with daridorexant 25 mg or 50 mg at either timepoint. IDSIQ, Insomnia Daytime Symptoms and Impacts Questionnaire; OSA**,** obstructive sleep apnea; SE, standard errors.

## **Figure S3: Efficacy of daridorexant on IDSIQ domain scores in participants with insomnia disorder and mild OSA, no OSA, and the overall phase 3 study population**

 
Forest plots of the placebo-corrected LS mean (95% CI) of IDSIQ sleepiness domain (A), IDSIQ mood domain (B), and IDSIQ alert/cognition domain (C) of the change from baseline to Month 1 and Month 3 in participants receiving daridorexant 25 mg or 50 mg. LS means were adjusted for the baseline value of the relevant parameter, age group (< 65 years; ≥ 65 years), treatment (daridorexant 50 mg; daridorexant 25 mg; placebo), visit (Month 1; Month 3), interaction of treatment by visit, and baseline by visit. The solid vertical line indicates the LS mean of the overall study population as reference; the dashed vertical line is the effect threshold. 
CI, Confidence Interval; Fav, favours; IDSIQ, Insomnia Daytime Symptoms and Impacts Questionnaire; LS mean, least square mean; OSA**,** obstructive sleep apnea.

## **Table S4: Subjective daytime sleepiness measured by Epworth Sleepiness Scale****©** **in participants with comorbid insomnia disorder and mild OSA** 

|  | **Daridorexant 50 mg** **(****n=53)** | **Daridorexant 25 mg** **(****n=53)** | **Placebo**  **(n=47)** |
| --- | --- | --- | --- |
| **ESS at baseline** |  |  |  |
| n | 53 | 53 | 47 |
| Mean (SD) | 6.8 (5.4) | 6.3 (5.2) | 6.0 (5.1) |
| >10 [n/N (%)] Run-in -2nd night | 13/52 (25.0) | 13/51 (25.5) | 8/42 (19.0) |
| **Change from baseline at Month 1** |  |  |  |
| n | 53 | 51 | 43 |
| Mean (SD) | -1.0 (3.3) | -1.4 (3.3) | -0.1 (3.0) |
| **Change from baseline at Month 3** |  |  |  |
| n | 50 | 50 | 45 |
| Mean (SD) | -1.5 (4.1) | -1.1 (4.1) | -1.6 (4.6) |

ESS©, Epworth Sleepiness Scale©; SD, standard deviation; OSA, obstructive sleep apnea.

## **Table S5: Adverse events in the safety analysis set of participants without OSA (n=270)**

|  | **Daridorexant 50 mg** **(n=255)** | **Daridorexant 25 mg** **(n=255)** | **Placebo** **(n=260)** |
| --- | --- | --- | --- |
| **Participants with ≥1 AE*** | 94 (36.9) | 101 (39.6) | 89 (34.2) |
| **AEs* of ≥3% in any group (preferred term)** |  |  |  |
| Nasopharyngitis | 19 (7.5) | 21 (8.2) | 18 (6.9) |
| Headache | 17 (6.7) | 12 (4.7) | 11 (4.2) |
| **AEs* leading to treatment discontinuation** | 3 (1.2) | 7 (2.7) | 8 (3.1) |
| **Participants with ≥1 SAE**^**†**^ | 1 (0.4) | 2 (0.8) | 7 (2.7) |
| Related to study treatment | 0 | 0 | 1 (0.4) |
| **Adjudicated AEs**^**‡**^ **(preferred term)** |  |  |  |
| Excessive daytime sleepiness | 0 | 3 (1.2) | 1 (0.4) |
| Sleep paralysis | 0 | 1 (0.4) | 0 |
| Hallucinations | 0 | 1 (0.4) | 0 |
| Suicidal ideation or self-injury | 0 | 0 | 0 |

Data are n (%). 
*Includes AEs that occurred during the double-blind treatment period with their preferred terms. 
^†^SAEs were reported during the double-blind study period up to 30 days after the end of treatment (or date of enrolment into the extension study)

^‡^Adjudicated AEs were reported during the double-blind study period up to 30 days after the end of treatment (or date of enrolment into the extension study) and were adjudicated blindly by an independent safety board.

AE, adverse event; OSA, obstructive sleep apnea; SAE, serious adverse event; TEAE, treatment-emergent adverse event.
